# Multi-locus models of genetic risk of disease

**DOI:** 10.1186/gm131

**Published:** 2010-02-02

**Authors:** Naomi R Wray, Michael E Goddard

**Affiliations:** 1 Genetic Epidemiology and, Queensland Institute of Medical Research, Herston Road, Brisbane, Queensland 4006, Australia; 2 Faculty of Land and Food Resources, University of Melbourne, Royal Parade, 3010 and Department of Primary Industries, Research Avenue, 3086, Melbourne, Victoria, Australia

## Abstract

**Background:**

Evidence for genetic contribution to complex diseases is described by recurrence risks to relatives of diseased individuals. Genome-wide association studies allow a description of the genetics of the same diseases in terms of risk loci, their effects and allele frequencies. To reconcile the two descriptions requires a model of how risks from individual loci combine to determine an individual's overall risk.

**Methods:**

We derive predictions of risk to relatives from risks at individual loci under a number of models and compare them with published data on disease risk.

**Results:**

The model in which risks are multiplicative on the risk scale implies equality between the recurrence risk to monozygotic twins and the square of the recurrence risk to sibs, a relationship often not observed, especially for low prevalence diseases. We show that this theoretical equality is achieved by allowing impossible probabilities of disease. Other models, in which probabilities of disease are constrained to a maximum of one, generate results more consistent with empirical estimates for a range of diseases.

**Conclusions:**

The unconstrained multiplicative model, often used in theoretical studies because of its mathematical tractability, is not a realistic model. We find three models, the constrained multiplicative, Odds (or Logit) and Probit (or liability threshold) models, all fit the data on risk to relatives. Currently, in practice it would be difficult to differentiate between these models, but this may become possible if genetic variants that explain the majority of the genetic variance are identified.

## Background

Complex genetic diseases are defined as those influenced by multiple genes and by environmental effects. In the past, individual genetic variants contributing to the risk of disease were usually not known, so the contribution of genes to disease was recognised through increased risk of disease in relatives of affected probands. Modeling allowed the genetic component of disease to be expressed as variance components and heritabilities. However, with the advent of genome-wide association studies (GWAS), individual genetic risk factors, or at least markers linked to them, are identifiable. This provides a description of the genetics in quite different terms to the traditional use of variance components. The new description is based on the frequency of individual risk alleles and their effect sizes expressed either as the relative risk or the odds ratio.

A clear picture is emerging as more and more results from GWAS are published about the effect sizes of individual loci that contribute to disease. For instance, allelic odds ratios at markers are typically estimated to be <1.5 and risk alleles can be the minor or major frequency allele. At present, there is little evidence of departure from a multiplicative model (on the observed disease risk scale) of disease [1], within and across loci, but this is based on combining only a limited number of markers and explaining only a small proportion of the genetic variance.

To reconcile the traditional description in terms of risk to relatives with the description based on individual risk loci, we need a model of how the risk loci combine to determine the total genetic risk for an individual person. Simple models are unlikely to be a true representation of complex diseases, but they allow us to explore the boundaries of possible genetic architectures that remain consistent with observed data. Several models are commonly used. Unfortunately the terms used to describe these models are confusing. For example, the terms 'additive' and 'multiplicative' can both be used to describe the same fundamental model because a multiplicative model on the observed disease risk scale (the 'risk scale') is equivalent to an additive model on the logarithm of the risk scale. Moreover, the multiplicative model can imply multiplicativity of allelic relative risks [2,3], or of odds ratios [4], or that risk alleles are needed at all loci in order to develop disease [5].

In this paper we show how the parameters for the individual risk loci (effect, allele frequency and number of loci) plus a model for combining the effects of individual loci determine the traditional parameters such as risk to relatives. The purpose of the paper is to compare the predictions made by different models and to determine which model(s) best fit the observed data. Before explaining the different models of genetic risk we first describe the genetic population parameters of recurrence risk to relatives.

### Recurrence risk to relatives

The genetic epidemiology of complex genetic diseases can be described in terms of the observable parameters of disease prevalence and relative risk to relatives of diseased probands (Table 1). Risks of disease in relatives provide an upper limit to the genetic component because common environmental factors may also increase risk to relatives. However, for the purposes of this paper we will assume risk to relatives is due to their genetic similarity. The recurrence risk for relatives of type *R *(*λ*_*R*_) is calculated as the ratio of the prevalence in the population of relatives of type *R *(*K*_*R*_) to the overall population prevalence (*K*), *λ*_*R *_≤ *K*_*R*_/*K*. As the maximum value for *K*_*R *_is 1 and the prevalence in monozygotic (MZ) twins of probands, *K*_*MZ*_, will be the highest of all relative types, there is a constraint that *λ*_*MZ *_= 1/*K*, so that higher values of *λ*_*MZ *_(and all *λ*_*R*_) are often observed for diseases of lower prevalence (Table 1). Despite being observable, the parameters *K *and *λ*_*R *_are subject to considerable sampling variance. For Table 1, we have tried, where possible, to take estimates from reviews or large studies, but large study samples simply do not exist for low prevalence disorders - for example, the *λ*_*MZ *_for ankylosis spondylitis [6] is based on only 27 MZ twin probands. Nonetheless, we can use these examples as a guide to assessing realistic scenarios for disease.

**Table 1:** Recurrence risk (*λ*_*R*_) to relatives (of type *R*) for several common complex genetic diseases ordered by prevalence (*K*)

Disease	Reference	*K*	*λ*_*MZ*_^a^	*λ*_*Sib*_^b^	*λ*_*OP*_		^***d***^	^***e***^	^***f***^	^***g***^
Major depression (population cohort)	[27]	0.24	2	1.3		0.32		3.3	1.2	0.34
Age related macular degeneration	[28,29]	0.12	4.7	2.1		0.50		3.4	1.1	0.64
Myocardial infarction	[30]	0.056	4.6	3.2		0.21		1.6	0.4	0.72
Breast cancer	[31]	0.036	4.1	2.2	1.9	0.12	1.3	2.6	0.8	0.37
Type II diabetes	[32]	0.028	10.4	3.5		0.27		3.8	0.8	0.58
Asthma	[33]	0.019	6.6	3.4		0.11		2.3	0.6	0.49
Rheumatoid arthritis	[34]	0.01	12.2	3.6		0.11		4.3	0.9	0.42
Bipolar disorder	[5]	0.01	60	7	7	0.60	1.0	10	1.2	0.70
Schizophrenia	[3]	0.0085	52.1	8.6	10	0.44	0.8	6.7	0.7	0.76
Type I diabetes	[35]	0.005	79	14		0.39		6.0	0.4	0.85
Multiple sclerosis	[36]	0.001	190	20		0.19	~1	9.9	0.5	0.68
Crohn's disease	[37]	0.001	600	64		0.60		10	0.1	1.00
Ankylosis spondylitis	[6]	0.001	630	82	79	0.63	1.0	7.8	0.1	1.00
Systemic lupus erythematosus	[38]	0.001		29	27		1.1			0.80
	[39,40]	0.0003	774	65		0.24		12	0.2	0.84

^a^The maximum prevalence for *K*_*MZ *_is 1, so *λ*_*MZ *_= *K*_*MZ*_*/K *is constrained to be ≤1/*K*. *λ*_*MZ *_was calculated from probandwise concordance rates *K*_*MZ *_and prevalence rates if *λ*_*MZ *_was not directly reported. ^b^Estimated from either sibling, dizygotic twin or first degree relative risks. ^c^ broad sense heritability on the risk scale (Equation 1). ^d^This ratio is expected to be 1 in the absence of dominance effects on the risk scale. ^e^This ratio is expected to be 2 under an additive model on the risk scale. ^f^This ratio is expected to be 1 under the unconstrained Risch model. ^g^Calculated from the estimates of *K *and *λ*_*Sib *_[41,42], constrained to a maximum of 1.

The risk to different classes of relatives (that is, *λ*_*R*_) depends on the magnitude of genetic variance components. The total genetic variance is traditionally decomposed into additive variance, dominance variance and various types of epistatic variance. The relationship between relative risks and variance components on risk scale was derived by James [7], who showed that the probability of disease in relatives of type *R *can be expressed as

with cov(*X*, *R*) the genetic covariance between the proband, *X*, and a relative, *R*. For individuals *X *and *R *we define *r *to be the relationship between them, *r *= 2 × Probability of identity by descent (IBD) of random alleles (that is, twice the ancestry or kinship coefficient) and *u *is the probability of both alleles being IBD at a locus, so that

where *V*_*A*(*k*)*D*(*l*) _denotes the genetic variance component with *k A *and *l D *terms [3,5,8,9]. So for *R *= MZ twin, *r *= 1, *u *= 1, then:

We use the '01' subscript to emphasize the observed zero-one (not diseased-diseased) risk scale of measurement. Therefore, an estimate of the broad sense heritability on the risk scale () is:(1)

since the phenotypic variance on the risk scale is  = *K*(1 - *K*). For the diseases listed in Table 1,  ranges from 0.11 to 0.63, but the heritability on this scale is not a normally reported statistic because of its dependence on disease prevalence. When the relatives are sibs, *R *= Sib, *r *= 1/2, *u *= 1/4, then:

When the relatives are parents or offspring, *R *= OP, *r *= 1/2, *u *= 0, then:

Therefore, *λ*_*Sib *_≥ *λ*_*OP *_since the former includes dominance terms; the magnitude of the ratio  reflects the relative importance of dominance effects. Often  (Table 1) and so dominance effects are considered to be negligible. This approximate equality also implies that common environmental effects between sibs is not different to that between parent and offspring, and, for many diseases, assuming common environmental effects are negligible seems plausible. Similarly, the ratio  is expected to be 2 under a model that contains only additive genetic variance; if individual risk loci combined additively on the risk scale, then only additive variance would be observed. This ratio is often greater than 2 (Table 1), implying that epistatic genetic variance on the risk scale is not negligible.

## Methods

### Genetic model

We define *K*, as before, as the disease prevalence and *g*_*x *_as the genetic risk (or probability) of disease of an individual given their multilocus genotype of *x *risk alleles out of a possible 2*n*, where *n *is the number of loci that contribute to the genetic variance of the disease; by definition E(*g*) = *K*. For simplicity, we will assume that all risk alleles have equal frequency, *p*, and equal relative risks, *τ*, compared to the non-risk (wild type allele). We discuss the implications of these assumptions later. We assume that all loci are independent and that each locus is biallelic and is in Hardy-Weinberg equilibrium so that the frequency of wild type, carrier and homozygous risk genotypes in the population are (1 - *p*)^2^, 2*p*(1 - *p*) and *p*^2 ^and *x *is distributed Binomial (2*n*, *p*), which approximates a normal distribution for *n *> ~5. We also assume random mating, no inbreeding and equal fertility of diseased and non-diseased individuals.

We consider three widely used genetic models of risk that are additive on some underlying scale. We assume that risk alleles act additively on the underlying scale both within a locus and between loci so that the critical contributor to genetic risk of disease is the number of risk alleles in an individual's multilocus genotype. We do not consider models that are additive on the risk scale as these were rejected by Risch [3] and confirmed in preliminary simulations as being unable to generate the patterns of recurrence risks to relatives observed for complex genetic diseases. After describing the disease risk models, we use numerical analysis and simulation to compare them. We compare the models to determine if they make the same predictions about observable recurrence risks and to investigate which model best fits the observed estimates.

#### Risch risk model

Additive on the log (risk) = log(*g*) scale: log(*g*_*x*_) = log(*f*_*n*_) + *x *log(*τ*)

Multiplicative on the risk (*g*) scale: *g*_*x *_= *f*_*n*_*τ *^*x*^

Under this model the relative risk of the risk allele compared to the other (wild-type) allele is *τ*, the homozygous risk genotype at each risk locus is *τ *^2 ^and the risks of the individual loci are multiplicative on the risk scale *g*_*x *_= *f*_*n*_*τ *^*x*^, where *f*_*n *_is the probability of disease in a person with only wild-type alleles at all *n *contributing loci and *f*_*n *_can be expressed explicitly as *f*_*n *_= *K*/(1 + *p*(*τ *- 1))^2*n *^[10]. This model of disease risk was introduced by Risch [3,11] and is the model that we [10] and others [2,12,13] have used in the prediction of genetic risk to disease from multiple loci. The multiplicative Risch model is attractive because of its mathematical properties, but an undesirable feature (often not apparent in the mathematical expressions) is that there is no constraint placed on *g*_*x*_, so that under some combinations of model parameters the probability of disease can have impossible values greater than 1 (that is, *g*_*x *_>1 for some *x*). This occurs when *x *≥ -ln(*f*_*n*_)/ln(*τ*) (after solving *f*_*n*_*τ *^*x *^= 1). We define the constrained Risch (CRisch) model to be the same as the Risch model except that *g*_*x *_is truncated to 1 [13]. In this case, if *K *is considered known, *f*_*n *_must be derived by numerically solving *K *= E(*g*) for *f*_*n *_assuming that *n*, *p *and *τ *are known.

#### Odds of risk model

Additive on the logit of risk scale: logit(risk) = log(*g*_*x*_/(1 - *g*_*x*_)) = log(*c*_*n*_*K*/(1 - *K*)) + *x*log(*γ*)

Multiplicative on the odds of risk scale: Odds = *g*_*x*_/(1 - *g*_*x*_) = *γ*^*x*^*c*_*n*_*K*/(1 - *K*) = *γ*^*x*^*C*_*n *_and so *g*_*x *_= *γ*^*x*^*C*_*n*_/(1 -*γ*^*x*^*C*_*n*_)

Under this model, *g*_*x*_/(1 - *g*_*x*_) is the odds of disease given the multilocus genotype and *C*_*n *_= *c*_*n*_*K*/(1 - *K*) is the odds of disease for an individual with all wild-type alleles at the *n *contributing loci, following Janssens *et al*. [4] and Lu and Elston [2]. The odds of disease without any information on multilocus genotype is *K*/(1 - *K*). Under this model the relative odds of risk of carriers and the homozygous risk genotypes are *γ *and *γ*^2^, where *γ *is the odds of the risk and where the *γ *are multiplicative on the odds of disease risk scale across loci. There is no explicit solution for *K *= E(*g*_*x*_) so that an explicit expression for *c*_*n *_cannot be derived. For given input parameters *c*_*n *_is derived by solving *K *= E(*g*_*x*_) numerically. Janssens *et al*. [4] used the approximation of *c*_*n *_= *c*_1_, but in preliminary studies we recognized that this approximation meant that the equality of E(*g*_*x*_) with the input (and key benchmark) parameter *K *was lost.

#### Probit of risk model or liability threshold model

Additive on an underlying liability scale: *u*_*x *_= (*x*-2*np*)*a*

Probit on the risk scale: 

Under this model we define *a *to be the effect of a risk allele on the underlying liability scale and *u*_*x *_is the genetic value on the underlying scale of an individual with *x *risk alleles, distributed about a mean of zero (since the mean number of risk alleles is 2*np*). Φ is the cumulative normal distribution function and *t *is a constant. The liability threshold model [14-16] assumes that liability to disease is normally distributed and that the presence of the disease arises if the liability exceeds a threshold, with the threshold positioned so that the proportion of the population that exceeds the threshold is equal to the population prevalence, *K*. The threshold, *t*, is derived from the inverse probability of the normal distribution, *t *= Φ^-1^(1 - *K*), Φ(*t*) = 1 - *K*; for example, if *K *= 0.05, *t *= 1.645. The model is parameterized in terms of variance components and heritability () on the underlying liability scale and can be scaled so that the phenotypic variance is 1. An individual's liability to disease is the sum of a genetic component (purely additive on this scale) distributed N(0, ) and an environmental component distributed N(0,1-). The number (that is, *n*) and frequency (that is, *p*) of risk alleles determine the value of *a*:

Although this model is often referred to as the liability threshold model, we will use the name 'Probit model' so that all three models are named on the risk scale.

### Relationship between relative risk (*τ*) and odds ratio (*γ*)

Under the Risch model, considering a single locus, the risk of the heterozygote is *τ *and the homozygote relative to the wild-type homozygote is *τ*^2^. Under this model the heterozygous odds ratio is:

Similarly, the homozygous odds ratio:

Therefore, OR_hom _>. In contrast, under the Odds model OR_het _= *γ*, OR_hom _= *γ*^2 ^and OR_hom_/ = 1. For example, *K *= 0.1, *p *= 0.1, *τ *= 2 under the Risch model, we can see that OR_het _= 2.49 and OR_hom_/OR^2^_het _= 1.13, which shows the Risch and Odds models to be quite different. However, under parameters more relevant to human disease, for example, *K *= 0.01, *p *= 0.1, *λ *= 1.05, then OR_het _= 1.0506 and OR_hom_/ = 1.00003. Hence, odds risks and relative risks are often used interchangeably because, at the single locus level, they are equivalent for practical purposes. However, under a multi-locus model, the differences between the models compound. Establishing a mathematical relationship between the multi-locus models is not tractable. So we have investigated this relationship by simulation.

### Comparison of models

One of the problems with comparing the models is to find a fair benchmark. We chose two parameters that are directly measurable in real populations for benchmarking models: disease prevalence and the effect size of a single risk allele. To achieve this benchmarking, four input parameters were needed for the Probit model from which all other variables are derived: disease prevalence, number of risk loci, frequency of risk allele and heritability on the liability scale (that is, *K*, *n*, *p *and ). To benchmark our comparisons, we set *τ*, the effect size of a single risk allele, to be equal to *g*_2*np*+1_*/g*_2*np*_with *g*_2*np*+1 _and *g*_2*np *_calculated from the Probit model. We use *τ *together with *K*, *n *and *p *as the input parameters for the Risch, CRisch and Odds models. Models are compared for the shape of the risk function, *g*_*x *_and on the broad sense heritability on the risk scale:(2)

where , and *q*_*x *_is the probability of an individual carrying *x *risk alleles.

To compare models we have used results from GWAS to inform us of realistic values of *τ*. We use *K *= 0.1, 0.01, 0.001, to be representative of common, complex genetic diseases and we use *K *= 0.5 to benchmark comparison at the most extreme prevalence rate and maximum phenotypic variance (*K*/(1 - *K*)) on the risk scale. Since the number of loci underlying complex diseases is an unknown, we use *n *= 100, 1,000, 10,000 since it is now considered unlikely that less than 100 loci will influence risk to common complex genetic diseases. We examined a range of *n*, *p *and , but have limited the results reported to situations that generate *τ *< 2. Although a few loci with *τ *> 2 have been identified (for example, for the late age of onset disorder, age related macular degeneration [17]), GWAS results suggest that the average *τ *will be less than this [18]. From simulation of 10^6 ^families over three generations, we calculate *λ*_*MZ*_, *λ*_*Sib*_, *λ*_*OP *_and the recurrence risk of disease in grandchildren of affected grandparents, *λ*_*OG*_. From these we calculate  (using equation 1) and  ≈ 4(*λ*_*OG *_- 1)*K*/(1-*K*), which is an estimate of narrow sense heritability that is less contaminated by non-additive variance than the estimate 2(*λ*_*OP *_- 1)*K*/(1-*K*). More detailed descriptions of the simulations are provided in Additional file 1.

## Results

### Risch versus constrained Risch model

In the unconstrained Risch model we found that the occurrence of the impossible probabilities of disease (*g*_*x *_> 1) had a significant impact on the results for some realistic combinations of parameters. For example, when *n *= 1,000, *K *= 0.1, *p *= 0.1, *τ *= 1.1, the mean number of risk alleles per person is 200 and *g*_*x *_> 1 when *x *> 232, which occurs with frequency 0.009. Despite the low frequency of occurrence, these extreme risks contribute disproportionately to the genetic variance and heritability. In this example, the heritability (calculated using equation 2) is 0.51, but falls to only 0.17 when these impossible risks are truncated to 1.

### Combined effect of *n*, *p *and *τ*

Results for a representative combination of parameters (*n *= 100, 1,000, 10,000, *K *= 0.1, 0.01, 0.001, *p *= 0.1, 0.3 and  = 0.5, 0.7; Additional file 2) show that although the broad sense heritability on the observed (that is, ; Equation 2) scale differs markedly between the Probit, CRisch and Odds models, there is little dependence on *n*, *p *and *τ *provided  is held constant. This is because, for a given , the parameters *n *and *p *control the variance contributed by each locus, so that when *n *is small, the effect size of each locus *τ *is necessarily high. These results imply that the key parameter in determining heritability on the risk scale is the total genetic variance rather than the variance at each locus. Consequently, the results are presented in terms of  (see 'Comparison of models' section above) because this allows translation to multiple combinations of *n*, *p *and *τ*.

### Shape of risk function and heritabilities on the risk scale

**Figure 1:**
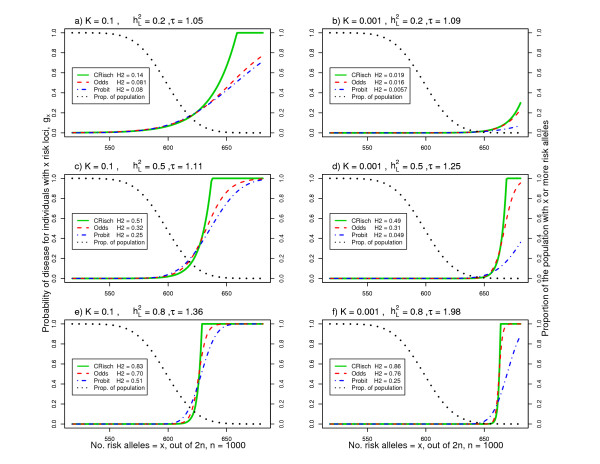
**Risk functions for the CRisch, Odds and Probit models using parameters relevant to human complex genetic diseases**. **(a-f) **Risk or probability (*g*_*x*_) of disease for an individual with *x *out of 2*n *risk alleles where the number of risk loci, *n *= 1,000 and the frequency of each risk allele, *p *= 0.3. The black dotted lines represent the proportion of individuals in the population who have *x *or more risk alleles. The parameters *n*, *p*, heritability on the underlying liability scale, , and disease prevalence, *K*, determine the relative risk of a single locus, *τ*. The legend lists the resulting broad sense heritability on the risk scale,  (H2 in the legend). The shape of the risk functions is achieved with other combinations of *n *and *p *for the same *K *and .

In Figure 1 we illustrate risk functions for combinations of parameters relevant to human complex genetic diseases. The x-axis is the number of risk alleles harbored by individuals in a population; theoretically, this can be between 0 and 2*n*, but in practice the number of risk alleles takes on the range 2*np *± 4√*2np*(1 - *p*), that is, 4 standard deviations about the mean. The number of risk alleles has an approximate normal distribution since the binomial distribution with large *n *tends to normality. In Figure 1, the black dotted line represents the proportion of individuals with *x *or more risk alleles. The 'S'-shaped curves are the risks or probability of disease given the number of risk loci, rising from *g*_*x *_= 0 to *g*_*x *_= 1. The positioning of this rise along the x-axis reflects the disease prevalence (that is, *K*) showing that, for low prevalence diseases, a greater number of risk alleles relative to the population mean is required for disease. The steepness reflects the broad sense heritabilities on the risk scale (that is, ) so that a steeper rise reflects a higher correlation between genotype and phenotype. Of these examples, only when  = 0.2 and *K *= 0.001 (Figure 1b) was there no need to constrain the Risch risk model as *g*_*x *_never reaches 1 even for the maximum values of *x *found in the population.

**Figure 2:**
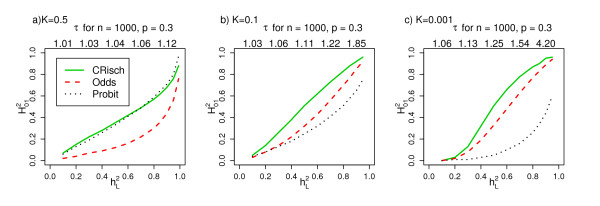
**Relationship between  for the CRisch, Odds and Probit models and , heritability on the underlying liability scale**. **(a-c) **For each , *τ *is estimated from the Probit model simulation and used as an input for the other models, so that all three models are benchmarked by *K *and *τ*. The shape of the relationship is not dependent on the choice of *n *and *p*; the *τ *when  = 0.1, 0.3, 0.5, 0.7 and 0.9 are listed above each graph when *n *= 1,000 and *p *= 0.3. From simulations of a single population of 10^6 ^individuals.

The relationship between  and *τ *or  is illustrated in Figure 2 and depends on both disease prevalence and model. Apparently small differences in the risk functions can have a big impact on the . For the Probit model  is a function of *K*, whereas for the CRisch and Odds models the dependence on *K *is of much less importance. This reflects the choice of benchmarking between the models. In the Probit model, the ratio  decreases as *x *(number of risk alleles) increases, whereas in the CRisch model this ratio is constant until the limit on probability of disease is reached. Therefore, the probability of disease rises more steeply with number of risk alleles for the CRisch model than the Probit model and this is more pronounced for rarer diseases when the difference between  at the average *x *and a high *x *is greater for the Probit model; the Odds model is intermediate.

**Figure 3:**
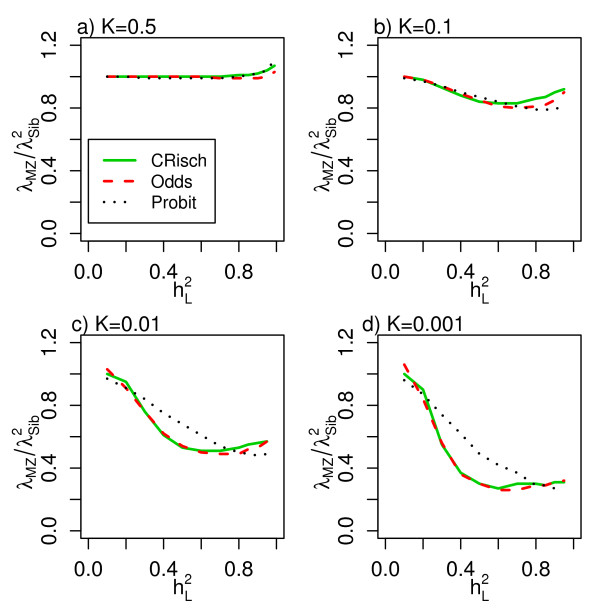
**Relationship between  and  for the CRisch, Odds and Probit models**. **(a-d) **Relationship for different disease prevalences (*K*).

Figure 3 presents the estimates of  across the full range of  and for different prevalences. Risch [3] predicted this relationship to be 1 under a multiplicative model. However, this relationship only holds when *K *= 0.5, or as  → 0 but becomes <<1 as *K *decreases and  → 1, a consequence of the need to constrain the probability of disease for an individual (*g*_*x*_) to a maximum value of 1. Values of *λ*_*MZ *_and *λ*_*Sib *_and the ratio  are presented for a range of scenarios (Table 2) to allow comparison with diseases listed in Table 1.

**Table 2:** Relative risks to relatives of affected individuals calculated within the stochastic simulation for Probit, CRisch and Odds models

		Probit			CRisch			Odds		
				
*K*		*λ*_*MZ*_	*λ*_*Sib*_		*λ*_*MZ*_	*λ*_*Sib*_		*λ*_*MZ*_	*λ*_*Sib*_	
0.1	0.1	1.3	1.2	0.99	1.4	1.2	1.00	1.3	1.1	1.00
0.1	0.5	3.2	1.9	0.87	5.6	2.6	0.84	3.9	2.1	0.85
0.1	0.7	4.7	2.4	0.81	7.6	3.0	0.83	6.0	2.8	0.80
0.1	0.95	7.8	3.1	0.82	9.7	3.2	0.92	9.3	3.2	0.90
0.01	0.1	1.9	1.4	0.97	2.4	1.5	1.00	1.7	1.3	1.03
0.01	0.5	13.0	4.4	0.68	51.7	9.9	0.53	34.8	8.1	0.54
0.01	0.7	26.6	7.0	0.54	76.8	12.3	0.51	62.3	11.3	0.49
0.01	0.95	67.3	11.7	0.49	97.0	13.0	0.57	94.6	12.9	0.57
0.001	0.1	2.8	1.7	0.96	4.0	2.0	1.00	1.2	1.1	1.06
0.001	0.5	54.8	10.5	0.49	516.5	41.6	0.30	342.5	34.0	0.30
0.001	0.7	157.8	20.6	0.37	796.8	51.4	0.30	638.5	49.5	0.26
0.001	0.95	599.8	47.5	0.27	989.9	57.6	0.30	968.6	55.9	0.31

is an input parameter for the Probit model. For each *τ *is estimated from the Probit model simulation and used as input to the CRisch and Odds model simulations.  is used as the benchmark as *τ *is dependent on *n*, *p *and *K*.

**Figure 4:**
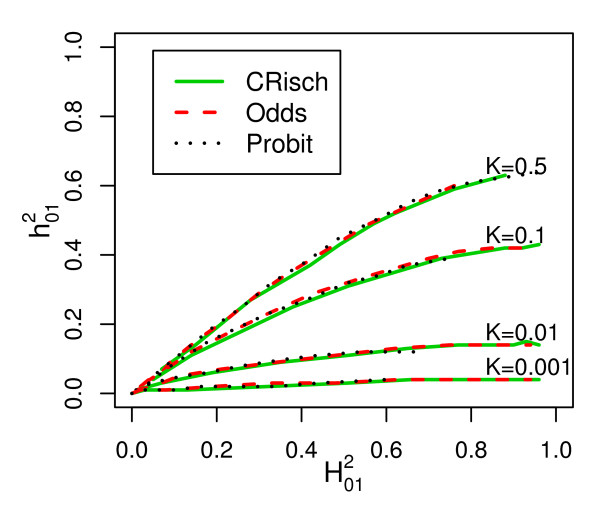
**Relationship between narrow sense (additive)  and broad sense heritability  on the risk scale for different disease prevalences (*K*)**. From simulations of a single population of 10^6 ^individuals, with  calculated as 4(*λ*_*OG *_- 1)*K*/(1-*K*) where *λ*_*OG *_is the recurrence risk of disease in grandchildren of affected grandparents and  calculated from Equation 2.

The relationship between  and  is almost the same for all models (Figure 4), confirming the similarity of the models on the risk scale. The maximum value of  is 0.64, which occurs as  → 1 when *K *= 0.5 as derived by Robertson (Appendix of Dempster and Lerner [14]). As *K *decreases or  increases the proportion of  that is additive declines so that, for diseases of prevalence ≤ 0.01 almost all of the heritability on the risk scale is explained by epistatic variance (as shown by the steep increase in the risk function [14]).

### Distinguishing between models based on risk to relatives

Although we assume that each risk locus has the same individual effect size, the models differ in the way that the effect sizes combine. In the CRisch model each additional risk allele multiplies probability of disease by the same amount until the number of risk alleles harbored reaches the limit of disease being certain, *g*_*x *_= 1. In contrast, the Odds and Probit models have 'built-in' constraints so that *g*_*x *_≤ 1, which means that each additional risk allele contributes proportionally less to the probability of disease. This effect can be seen in Figure 1 where the risk function is steepest for the CRisch model and least steep for the Probit model with the Odds model usually in between the other two. The steeper the risk function the higher the broad sense heritability , so this is usually highest for the CRisch model and least for the Probit model. This effect of the risk function on heritability on the risk scale also applies to the narrow sense heritability, , so the relationship between the two remains constant (Figure 4). The similarity of the models on the risk scale is not perfect as shown by differences in  in Figure 3. However, if this ratio is graphed against a function of observable parameters, such as  instead of , the differences between models are small (Additional file 3) and could not be demonstrated in practice given the sampling errors of the parameters. Thus, the three models could not be distinguished using only traditional data, that is, recurrence risk of relatives.

### Distinguishing between models based on relative risks of individual loci, *τ*

If we identify one or more loci affecting a disease, we can directly observe the risk in people carrying different numbers of risk alleles and compare this with the model predictions. The numerical example in the 'Relationship between *τ *and *γ *' section shows that, for a single locus, the models do make different predictions when *τ *values are large but not when they are small, as is expected to be the usual case. However, even for small *τ *values the models differ when all risk loci are included. To obtain the same heritability on the risk scale, the models required different effect sizes (*τ*) of associated variants (Figure 2). Similarly, by comparing Tables 1 and 2, we can see that combinations of observed *λ*_*MZ *_and *λ*_*Sib *_correspond to a much lower *τ*, which translates to a lower heritability on the liability scale under the CRisch or Odds model compared to the Probit model. For example, for a disease with prevalence *K *= 0.01, *λ*_*MZ *_= 52, *λ*_*Sib *_= 10 (parameters representative of schizophrenia), the *τ *for *n *= 1,000 loci each with risk allele frequency *p *= 0.3 were 1.19, 1.26 and 1.41 for the CRisch, Odds and Probit models, respectively. However, only if it is possible to identify the majority of the risk variants will it be possible to differentiate between the models in practice.

Another way to look at this difference between the models is that, for a given value of *λ*_*MZ *_(or *λ*_*Sib*_) and *τ *and *p*, a higher value of *n *is required for the Probit model than for the CRisch model. This means that a given risk locus with observed *τ *and *p *explains a smaller proportion of the risk to relatives under a Probit model than under a CRisch model. Or equally, it means that the CRisch models generate higher risks to relatives in our benchmarked comparisons - for example, when *K *= 0.01, *n *= 1,000, *p *= 0.3, *τ *= 1.2 and  = 0.5, *λ*_*MZ *_for the CRisch, Odds and Probit models were 52, 35 and 13, respectively; the *λ*_*Sib *_for the same models were 10, 8 and 4, respectively. If risk loci are identified that account for a significant proportion of the sibling risk, then it may be possible to test which model better fits observed data, but this will require a large number of families to be genotyped for the risk loci.

## Discussion

With the advent of GWAS we are gaining a clearer understanding of the genetic architecture of common complex diseases. Empirical evidence suggests an architecture of many genetic loci with many variants of small effect. Interest in genomic profiling, the use of a genome-wide markers to predict genetic disease risk, is growing (for example, [19,20]), as is the establishment of companies offering profiling services. The prediction of disease risk from many risk loci or markers requires a model that combines the effects of these loci and the choice of this model is the topic of this paper.

### Total variance of risk loci is the driving force

We chose two parameters that are directly measurable in real populations for benchmarking models: disease prevalence (that is, *K*) and the effect size of a single risk allele (that is, *τ*). We recognized that many combinations of the number of loci (that is, *n*) allele frequency (that is, *p*) and *τ *were consistent with the same heritability on the underlying scale in the Probit model (that is, ) and that the predictions of all the models were insensitive to the exact combination of *n*, *p *and *τ *provided  was held constant. Therefore, we have compared the models while holding constant *K *and . In Figures 1 and 2 we present results for *n *= 1,000 and *p *= 0.3, to provide some comparison to empirical estimates of *τ*. Since the distribution of genetic risk of disease in a population is driven by total genetic variance rather than the variance contributed by each locus, it is unlikely that relaxing the restriction of equal allele frequencies and effect sizes will impact the results; this is consistent with the results of other studies [4,10,21].

Although we show that the unconstrained Risch model is not a practical model, its mathematical tractability can still provide valuable insight into our understanding of the factors influencing genetic risk. We show (Additional file 4) that the scaled contribution to the genetic variance on the risk scale by each risk allele (*v*) is a function of *p *and *τ*, *v *= *p*(1 - *p*)(*τ *- 1)^2^/[1 + *p*(*τ *- 1)]^2 ^and the total genetic variance on this scale is proportional to *nv*. For small values of *τ *(that is, *τ*; → 1), *nv *≈ *np*(1 - *p*)(*τ *- 1)^2^, which can be used to derive the proportion of genetic variance explained by one locus.

### Rejection of simple additive and simple multiplicative models on the risk scale

Risch [3], using schizophrenia as an example, was the first to show that recurrence risk to relatives in complex diseases is better explained by a multiplicative than an additive model of gene action on the risk scale because (*λ*_*MZ *_- 1)/(*λ*_*sib *_- 1) >2 as shown in Table 1. In preliminary simulations (not reported) we confirmed that additivity on the risk scale of all risk loci simply could not produce the steep rise in probability of disease (Figure 1) necessary to achieve the disease prevalences and recurrence risks to relatives typical of complex diseases. In contrast, Slatkin [13], under his thesis of exchangeable models, demonstrated that an additive model on the risk scale could explain complex disease. However, to achieve the steep rise in disease risk, he imposed stringent constraints, so that the additive effect of risk alleles only occurred in the (very narrow) range of the number of risk alleles associated with the steep rise in probability of disease. Outside this range probability of disease was either zero or 1. In this way, the shape of the risk function is similar to the models that are multiplicative on the risk scale.

Other theoretical studies have used the Risch model [2,13], the CRisch model [13], the Odds model [4] and the Probit model [22]. Although there is a generally accepted dogma that these models are similar, in trying to compare studies it is important to know if any differences are a function of the choice of risk model. In a previous study [10] we made derivations under the Risch model and for the parameter combinations considered the probability of disease being greater than 1 was rare. However, in this study, where we have considered the full range of parameters, we have recognized that under the unconstrained Risch model, individuals for whom probability of disease is greater than 1 (*g*_*x *_>1) make a huge contribution to the genetic variances.

Risch [3] investigating schizophrenia and Brown *et al*. [6] studying ankylosing spondilitis recognized that the observed ratio  was less than one, whereas this ratio is expected to be 1 under the Risch model [3]. The sampling variance on estimates of recurrence rates is high and so the greater consistency with multiplicative rather than additive models (risk scale) was their main conclusion. However, by looking at a range of complex diseases (Table 1) there is consistent evidence that  is less than 1, particularly for low prevalence diseases. These observed ratios are consistent with our simulation results, which show that under the CRisch, Odds and Probit models, the ratio  only as *K *→ 0.5 and  → 0, but under parameters typical of common complex genetic diseases , particularly as *K *→ 0 and  → 1. The mathematical tractability of the Risch model has often made it the method of choice in theoretical studies and the equality  has been used to underpin predictions (for example, see the Supplement of Clayton [23]); in the mathematical expressions the impact of not constraining the probability of disease to be less than 1 is not obvious, but it is because of this important constraint that equality  is often much less than 1.

Therefore, we conclude that the unconstrained Risch model is simply not realistic, particularly for parameters typical of human complex disease (K < 0.1 and  > 0.5), so here we have made comparisons on the more realistic constrained (CRisch) model.

### Differences between the models unlikely to be detectable in practice

Since we reject the additive and Risch models, we concentrate on the comparison of the CRisch, Odds and Probit models. We chose to compare models with two fixed benchmarks, disease prevalence and effect size of an individual risk allele, taken at the average number of risk alleles (that is, *τ*). Under this benchmarking, the probability of disease associated with carrying the minimum number of alleles in the population differs between models, but in all models this will be very close to zero given the number or risk loci now expected to contribute to complex genetic disease. Although we assume that each risk locus has the same individual effect size, the models differ in the way that the effect sizes combine. For example, a given risk locus with observed *τ *and *p *explains a smaller proportion of the risk to relatives under a Probit model than under a CRisch model However, we conclude that for all operational purposes, in the foreseeable future, it is unlikely that we will be able to distinguish between the models either on the basis of recurrence risks to relatives or on the basis of estimates of effect sizes of risk loci. Slatkin [13] also compared the CRisch and Probit models and benchmarked on a range of parameters. Our results are complementary to, and consistent with, his, although direct comparison is prevented by his models distinguishing between heterozygotes and homozygotes at each locus, so that the multiplicativity of risk alleles was only between loci and not within loci. Inability to distinguish between multi-locus risk models on the basis of recurrence risks is perhaps not surprising given that Smith [24] was unable to distinguish between more extreme models on this basis. Ability to distinguish between the models is only possible in the very tail of the risk curve and would only be achievable if genomic profiles could be constructed using measured variants that accounted for the totality of the genetic variance. If this were possible, sets of individuals could be identified with high predicted risk and the proportion succumbing to disease could be measured and compared to the proportion expected under different models. Such hypothetical scenarios at present seem unattainable.

### Each individual carries a unique portfolio of risk loci

From Figure 1 it becomes clear that when there are many risk loci contributing to disease each of small effect, that all individuals in the population necessarily carry a large number of risk alleles. For example, when 1,000 loci with risk alleles of frequency 0.1 underlie a complex disease, all individuals in the population carry at least 150 risk alleles, an average individual carries 200 risk alleles and, when disease prevalence is low and heritability is high, most of those with disease carry 230 to 250 risk alleles. Since, in this example, there is a total of 2,000 risk alleles, each individual will carry their own unique portfolio, which could underlie the phenotypic heterogeneity typical of many complex diseases.

### Large amounts of epistasis on the risk scale despite additivity on underlying scales

Our results show that additivity of individual genetic variants on some underlying scale can convert to, sometimes considerable, non-additive genetic variance on the risk scale, particularly when the disease prevalence is low. These results are not new and were presented by Dempster and Lerner [14], but are sometimes overlooked. Human diseases usually have prevalences of less than 0.1, in which case the majority of the genetic variance on the risk scale is epistatic. These results imply that the models underpinning GWAS already account for one type of gene-gene interaction, if each *τ *could be estimated without error. Likewise, our usual models also imply genotype-environment interaction on the risk scale because the effect of an environmental factor is greater in people with higher genetic risk. Our definition of epistasis is one of statistical interaction; the extent to which statistical interaction relates to biological or functional interaction has been much debated (see [25] for a review) and will not become clear until more of the genetic variance can be explained by identified genomic variants.

### True versus estimated *τ*

We set out to benchmark models on the basis of two observable parameters, disease prevalence (that is, *K*) and the effect size of a single risk allele (that is, *τ*). In building the models we have assumed that the true *τ *is known and have defined it as the effect of a single risk locus in the background of the average number of risk loci. However, the estimates of *τ *made from experimental data may be quite different to these true values. If the genotypes at all risk loci were known and a complete model was fitted to the data, then the correct estimate of *τ *would be obtained (within experimental sampling error). In practice, however, usually only the effect of a single risk locus is included in the statistical model and under these circumstances we will estimate the effect of an extra risk allele averaged across all background genotypes rather than the effect at the mean background genotype. The effect of this may be dependent on the true way in which loci combine to influence risk of disease, which, of course, is unknown. Under the CRisch model of Figure 1a, all individuals with >650 risk alleles get the disease, so above 650 risk alleles there is no effect of an extra risk allele. Conversely, below 650 risk alleles each extra risk allele increases the probability of disease by *τ*. The experimental estimate will be a weighted average of these two estimates (zero and *τ*). In practice, therefore, variants detected with small relative risk may reflect greater biological importance than might otherwise be inferred. Under the Probit model the *τ *calculated at the average number or risk loci is  whereas the *τ *estimated when a single risk locus is in the statistical model is Φ(a-t)/Φ(-*t*) because then all other risk loci are part of the residual variance in liability and so the residual variance approaches the phenotypic variance, which is 1.0.

Comparison of the models in practice is difficult and distinguishing between them may be impossible, especially if the true *n *is large and the true *τ *is small. Since we have demonstrated that the models are difficult to differentiate, the use of the Probit model, which has mathematical tractability and a known relationship between the estimates of *τ *in different genetic backgrounds, is likely to be the model of choice. The estimated variance on the liability scale explained by a locus with estimated effect size  is [26], so that the estimated effect on the liability scale is , where *i *is the mean liability of the diseased group, *i = z/K*, where *z *is the height of the normal curve at the threshold *t*.

### Limitations

The true genetic architecture (in terms of number, frequency and effect size of risk variants and the way in which they combine) is unknown and may be quite different for the different diseases listed in Table 1. For simplicity, we have described disease in terms of affected/unaffected, ignoring time-dependent onset, and we have ignored phenotypic heterogeneity (which may reflect genetic heterogeneity) in the definition of disease status and other real-life complications. In principle, our approach could reflect any definition of disease if the genetic epidemiology and genetic risk variants can be defined - for example, early and late onset disease may be considered as different diseases - but despite this any simple model is likely to be a poor representation of disease. None of the models we have considered are likely to be the true model, but since they can all generate recurrence risks consistent with complex genetic diseases (given the right combination of parameters), they can give useful insight until empirical data provide evidence for them to be rejected. These simple models provide some boundaries, demonstrating some properties that must be upheld by the true genetic architecture in order to be consistent with observed data.

## Conclusions

In this paper we set out to compare different models that combine the effects of multiple risk loci into an overall genetic risk. We conclude that a model that is additive or multiplicative on the risk scale across all loci is incompatible with the observed recurrence risks to relatives. The constrained multiplicative (CRisch), Odds and Probit models are all compatible with the observed data and, in fact, it is difficult to distinguish between them when the relative risk at an individual locus is small. Importantly, we show that the unconstrained multiplicative (Risch) model, often used in theoretical studies because of its mathematical tractability, is not a realistic model as impossible probabilities of disease are implied. Specifically, the multiplicative Risch model generates a relationship of  = 1, but we have demonstrated that this not possible under many disease scenarios and occurs in the theoretical derivation because probabilities of disease are not constrained and can exceed 1. We have demonstrated that under more realistic models in which probabilities of disease are constrained to 1, the ratio  is often much less than 1, a result that is consistent with empirical estimates from a range of diseases. Finally, we conclude that it will only be possible to distinguish between the CRisch, Odds and Probit models in practice if genetic risk profiles are able to reconstruct the majority of the known genetic variance; this is unlikely for the foreseeable future.

## Abbreviations

CRisch: constrained Risch; GWAS: genome-wide association study; MZ: monozygotic. *γ*: odds of disease for risk allele compared to wild-type allele; *λ*_*MZ*_: recurrence risk of disease in monozygotic twins of diseased individuals; *λ*_*OG*_: recurrence risk of disease in grandoffspring of diseased grandparents; *λ*_*OP*_: recurrence risk of disease in offspring of diseased parents; *λ*_*R*_: recurrence risk of disease in relatives of diseased individuals for relatives of type *R*; *λ*_*Sib*_: recurrence risk of disease in sibs of diseased individuals; *τ*: the risk (probability) of disease of a risk allele relative to the other (wild-type) allele for a single locus (for the unconstrained Risch model *τ *= *g*_*x*+1_/*g*_*x *_for all *x *= 0, 2*n *- 1); *a*: additive effect size of each risk allele on the liability scale in Normal standard deviation units; *f*_*n*_: probability of disease in a person with wild-type alleles only at all *n *contributing loci; *g*_*x*_: the genetic risk (or probability) of disease of an individual given their multilocus genotype of *x *risk alleles; : narrow sense (that is, additive genetic) heritability on the risk scale; : heritability on the liability scale, on this scale all genetic variance is additive; : broad sense (that is, total genetic) heritability on the risk scale - on this scale the phenotype, disease, is either not diseased (0) or diseased (1); *K*: disease prevalence in a population; *K*_*R*_: disease prevalence in relatives of diseased individuals for relatives of type *R*; *n*: the number of loci that contribute to the genetic variance of the disease; *p*, frequency of risk allele; *t*: threshold truncating proportion *K *in the right-hand tail of the normal distribution; *x*: number of risk alleles harbored by an individual, between 0 and 2*n*.

## Competing interests

The authors declare that they have no competing interests.

## Authors' contributions

NRW and MEG together devised the study, interpreted the results and wrote the manuscript. NRW conducted all simulations. MEG derived the 'Variance components on the risk scale using the unconstrained risk model' in the Additional files. Both authors read and approved the final manuscript.

## Supplementary Material

Additional file 1 **A detailed description of simulations**. A detailed description of simulations. Click here for file

Additional file 2**A table showing broad sense heritabilities on the disease risk scale**. A table showing broad sense heritabilities on the disease risk scale,  (Equation 2), for different combinations of disease prevalence, *K*, number of risk loci, *n*, risk allele frequency, *p*, heritability on the liability scale, , and risk of a single risk allele compared to the non-risk allele, *τ*.Click here for file

Additional file 3**Figure showing the relationship between  and  for the CRisch**. A figure showing the relationship between  and  for the CRisch, Odds and Probit models and different disease prevalences (*K*).Click here for file

Additional file 4 **A PDF document providing variance components on the risk scale using the unconstrained risk model**. A PDF document providing variance components on the risk scale using the unconstrained risk model. Uses the mathematical tractability of the unconstrained Risch model to examine the contribution of each risk allele to genetic variance on the risk scale. Click here for file
