# Supplementary material for: Multi-locus models of genetic risk of disease
Source: Genome Med. 2010 Feb 2;2(2):10. doi: 10.1186/gm131 (PMC2847701; doi:10.1186/gm131)
Supplement: Additional file 1 — A detailed description of simulations. A detailed description of simulations. [file gm131-S1.PDF]

Detailed description of simulations

Deterministic simulation (direct numerical analysis)

For each model we evaluated the probability of disease (g_x) as described in the main paper using direct numerical evaluation, calculating the probability (q_x) of an individual having (or equally the proportion of the population with) $x = 0, 1.. 2n$ risk loci, since $x \sim B(2n,p)$. In the Odds model $\gamma = \tau(1-f_n)/(1-\tau f_n)$.

Stochastic simulation

We use stochastic simulation to allow comparison models on the measures of risk rates in relatives and narrow sense heritability on the risk scale (h^2_{01}). Input parameters are K, n, p and h^2_L or τ, but based on the results from the deterministic simulation we consider only n =1000 and p = 0.3. For the Risch and Odds models we simulate the alleles at each locus for individuals over three generations for $N = 10^6$ families. Alleles of founders are sampled as B(2, p). Each child receives one allele sampled at random from each parent. For each individual the number of risk alleles x is calculated. The probability of disease for the Risch model is: $g_x = f_n \tau^x$, and Odds model: $g_x = Kc_n\gamma^x / [1 + K(c_n\gamma^x - 1)]$ with f_n and c_n derived iteratively as in the deterministic simulation. For each individual, we sampled w ~U(0,1) and if $g > w$, the individual was considered affected, otherwise not affected. The disease status of an MZ twin was calculated in the same way by drawing a second random number from the uniform distribution.

For the Probit model, the input parameters were K and h^2_L calculated as for the deterministic model and assumed a normal distribution of genetic effects on the underlying liability scale. The results from the deterministic simulations had shown that the results were independent of n and p and the variance contributed by each

locus, but were dependent only on the total variance contributed by all loci, parameterised through h_L^2. The genetic (u) and phenotypic (y) risk of disease on the liability scale was simulated as $y = u + e$ where $u \sim N(0, h_L^2)$ for founders and $u = ½u_{dad} + ½u_{mum} + u_{mend}$ for children where the mendelian segregation term $u_{mend} \sim N(0,½h_L^2)$. The environmental effect $e \sim N(0,1\text{-}h_L^2)$. The underlying risk phenotype of an MZ twin was simulated by drawing a new e for an individual. Individuals were considered affected if $y > \Phi^{-1}(1\text{-}K) = t$.

From these simulations we could calculate λ_{MZ}, λ_{Sib}, λ_{OP} and the recurrence risk of disease in grandchildren of affected grandparents, λ_{OG}. From these we calculate H_{01}^2 (equation 1) and $h_{01}^2 \approx 4(\lambda_{OG} - 1)K/(1-K)$ which is an estimate of narrow sense heritability that is less contaminated by non-additive variance than the estimate $2(\lambda_{OP} - 1)K/(1-K)$. We used the results from the deterministic simulation to check results from the stochastic simulation (the deterministic results are a subset of the stochastic results) and in the Results section we do not distinguish between them.
