# Supplementary material for: Multi-locus models of genetic risk of disease
Source: Genome Med. 2010 Feb 2;2(2):10. doi: 10.1186/gm131 (PMC2847701; doi:10.1186/gm131)
Supplement: Additional file 2 — A table showing broad sense heritabilities on the disease risk scale. A table showing broad sense heritabilities on the disease risk scale, (Equation 2), for different combinations of disease prevalence, K, number of risk loci, n, risk allele frequency, p, heritability on the liability scale, , and risk of a single risk allele compared to the non-risk allele, τ. [file gm131-S2.DOCX]

Broad sense heritabilities on the disease risk scale, $H_{01}^{2}$ (equation 2) for different combinations of disease prevalence, *K*, number of risk loci, *n*, risk allele frequency, *p*, heritability on the liability scale,$h_{L}^{2}$ and risk of a single risk allele compared to the non-risk allele, *τ* .

|  |  |  |  |  |  | $H_{01}^{2}$ |  |
| --- | --- | --- | --- | --- | --- | --- | --- |
| *K* | *n* | *p* | $h_{L}^{2}$ | * * | Probit | CRisch | Odds |
| 0.001 | 1000 | 0.1 | 0.7 | 1.93 | 0.15 | 0.76 | 0.62 |
| 0.001 | 1000 | 0.3 | 0.7 | 1.54 | 0.15 | 0.77 | 0.62 |
| 0.001 | 10000 | 0.1 | 0.7 | 1.23 | 0.15 | 0.77 | 0.62 |
| 0.001 | 10000 | 0.3 | 0.7 | 1.15 | 0.15 | 0.77 | 0.62 |
| 0.01 | 1000 | 0.1 | 0.7 | 1.65 | 0.26 | 0.76 | 0.61 |
| 0.01 | 1000 | 0.3 | 0.7 | 1.39 | 0.26 | 0.76 | 0.62 |
| 0.01 | 10000 | 0.1 | 0.7 | 1.17 | 0.26 | 0.77 | 0.62 |
| 0.01 | 10000 | 0.3 | 0.7 | 1.11 | 0.26 | 0.77 | 0.62 |
| 0.1 | 100 | 0.3 | 0.7 | 1.83 | 0.41 | 0.72 | 0.55 |
| 0.1 | 1000 | 0.1 | 0.7 | 1.35 | 0.41 | 0.73 | 0.56 |
| 0.1 | 1000 | 0.3 | 0.7 | 1.22 | 0.41 | 0.73 | 0.56 |
| 0.1 | 10000 | 0.1 | 0.7 | 1.10 | 0.41 | 0.73 | 0.56 |
| 0.1 | 10000 | 0.3 | 0.7 | 1.06 | 0.41 | 0.73 | 0.56 |
| 0.001 | 1000 | 0.1 | 0.5 | 1.40 | 0.05 | 0.49 | 0.32 |
| 0.001 | 10000 | 0.1 | 0.5 | 1.11 | 0.05 | 0.49 | 0.32 |
| 0.001 | 1000 | 0.3 | 0.5 | 1.25 | 0.05 | 0.49 | 0.31 |
| 0.001 | 10000 | 0.3 | 0.5 | 1.07 | 0.05 | 0.49 | 0.32 |
| 0.01 | 1000 | 0.1 | 0.5 | 1.30 | 0.12 | 0.51 | 0.34 |
| 0.01 | 10000 | 0.1 | 0.5 | 1.09 | 0.12 | 0.51 | 0.34 |
| 0.01 | 100 | 0.3 | 0.5 | 1.71 | 0.12 | 0.50 | 0.33 |
| 0.01 | 1000 | 0.3 | 0.5 | 1.19 | 0.12 | 0.51 | 0.34 |
| 0.01 | 10000 | 0.3 | 0.5 | 1.06 | 0.12 | 0.51 | 0.34 |
| 0.1 | 100 | 0.1 | 0.5 | 1.64 | 0.25 | 0.50 | 0.31 |
| 0.1 | 1000 | 0.1 | 0.5 | 1.18 | 0.25 | 0.51 | 0.32 |
| 0.1 | 10000 | 0.1 | 0.5 | 1.05 | 0.25 | 0.51 | 0.32 |
| 0.1 | 100 | 0.3 | 0.5 | 1.39 | 0.25 | 0.50 | 0.31 |
| 0.1 | 1000 | 0.3 | 0.5 | 1.11 | 0.25 | 0.51 | 0.32 |
| 0.1 | 10000 | 0.3 | 0.5 | 1.03 | 0.25 | 0.51 | 0.32 |

Input parameters for all models are *K, n* and *p*. $h_{L}^{2}$is an input parameter for the Probit model only.*  * is an input parameter for the CRisch and Odds model simulations and is calculated as* *at the average number of risk loci in the Probit model simulations.
