# Supplementary figures and images for: Multi-locus models of genetic risk of disease
Source: Genome Med. 2010 Feb 2;2(2):10. doi: 10.1186/gm131 (PMC2847701; doi:10.1186/gm131)

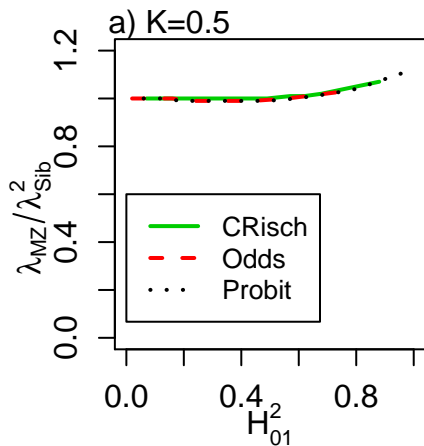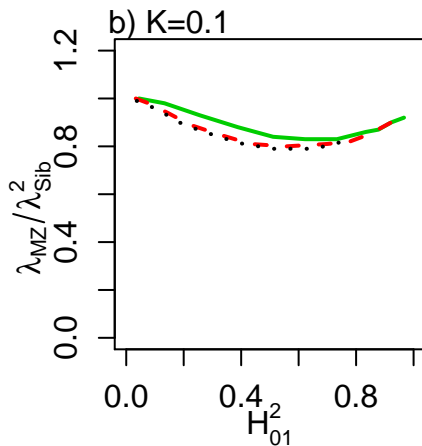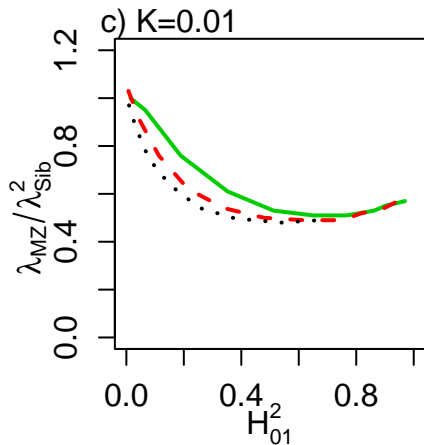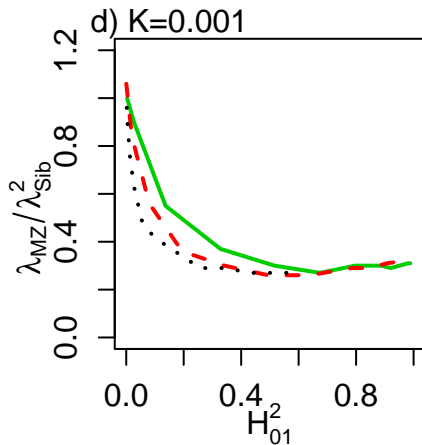

Supplement: Additional file 3 — Figure showing the relationship between and for the CRisch. A figure showing the relationship between and for the CRisch, Odds and Probit models and different disease prevalences (K). [file gm131-S3.PDF]
