# Supplementary material for: Multi-locus models of genetic risk of disease
Source: Genome Med. 2010 Feb 2;2(2):10. doi: 10.1186/gm131 (PMC2847701; doi:10.1186/gm131)
Supplement: Additional file 4 — A PDF document providing variance components on the risk scale using the unconstrained risk model. A PDF document providing variance components on the risk scale using the unconstrained risk model. Uses the mathematical tractability of the unconstrained Risch model to examine the contribution of each risk allele to genetic variance on the risk scale. [file gm131-S4.PDF]

Variance components on the risk scale using the unconstrained risk model.

Following our previous derivations [10], the total genetic variance on the risk scale can be expressed as $V_{G_{01}} = \text{var}(f_n \tau^x) = \text{E}[(f_n \tau^x)^2] - (\text{E}[f_n \tau^x])^2 = f_n^2[(1 + p(\tau^2-1))^{2n} - (1 + p(\tau-1))^{4n}]$. Using $f_n = K/(1+p(\tau-1))^{2n}$

$$V_{G_{01}} = K^2[(1+v)^{2n} - 1],$$

where $v = p(1-p)(\tau-1)^2 / [1+ p(\tau-1)]^2$ and parameterises the contribution to the genetic variance of each risk allele. Additive ($V_{A_{01}}$) and dominance ($V_{D_{01}}$) variance are obtained by summing the single locus variances (which are given in [3,10,11]). Hence, $V_{A_{01}} = 2nvK^2$ and $V_{D_{01}} = nv^2K^2$. By definition, the total amount of epistatic variance is $V_{G_{01}} - V_{A_{01}} - V_{D_{01}}$. The total genetic variance can also be written as $V_{G_{01}} = K^2 [(1+2v+v^2)^n - 1]$. A binomial expansion of the term $(1+2v+v^2)^n - 1$ gives terms which correspond to all additive and non-additive variance components:

$$K^2 [(1+2v+v^2)^n - 1] = K^2[2nv + nv^2 + 0.5n(n-1)(4v^2 + 4v^3 + v^4) + \dots]$$

$V_{A_{01}}$	$V_{D_{01}}$	$V_{AA_{01}}$	$V_{AD_{01}}$	$V_{DD_{01}}$	+ etc.
Single locus terms		Two locus terms			> 2 locus terms

With many loci, (λ-1) is small, so v is small (<1) and v^2 is even smaller. Consequently, the dominance components become negligible. The epistatic *AA*, *AAA*, etc. components do not become negligible, however, because they are multiplied by higher powers of n than the dominance components. For large n, $V_{G_{01}} \approx K^2(e^{2nv} - 1)$. Therefore, the proportion of genetic variance that is non-additive depends on the size of $2nv$ which is the scaled additive variance on the risk scale. The ratio of the additive to total genetic variance on the risk scale is $V_{A_{01}} / V_{G_{01}} \approx 2nv/(e^{2nv} - 1)$. Hence, paradoxically, if $2nv$ and therefore the additive variance is large, then epistatic variance is even larger and most of the genetic variance on the risk scale is non-additive.
